# Silent signals: how *N*-acyl homoserine lactones drive oral microbial behaviour and health outcomes

**DOI:** 10.3389/froh.2024.1484005

**Published:** 2024-12-05

**Authors:** Zelda Ziyi Zhao, Lifeng Guo, Wenwen Shan, Chun Hung Chu, Jing Zhang

**Affiliations:** ^1^ Faculty of Dentistry, The University of Hong Kong, Hong Kong, Hong Kong SAR, China; ^2^ College & Hospital of Stomatology, Key Laboratory of Oral Diseases Research of Anhui Province, Anhui Medical University, Hefei, China

**Keywords:** acyl-homoserine lactones, quorum sensing, microbial interaction, biofilm, oral health, quorum quenching acyl-homoserine lactones, quorum quenching

## Abstract

**Background:**

*N*-acyl homoserine lactones (AHLs) are small signalling molecules predominantly secreted in Gram-negative bacteria.

**Objective:**

The aim is to provide a comprehensive overview of AHLs in oral health.

**Methods:**

Two independent researchers conducted a systematic search of English language publications up to 30 June 2024 in PubMed, Scopus and Web of Science. They screened the title and abstract to retrieve and map out relevant studies on AHLs in oral health, in order to identify key concepts, gaps in knowledge, and areas for further research.

**Results:**

This study identified 127 articles and included 42 articles. These studies identified AHLs in human oral samples like saliva, dental plaque, tongue swabs, and dentin caries. The studies also found that AHLs regulate cell-to-cell communication of bacteria (quorum sensing) in mature biofilm fostering the production of virulence factors that damage the immune system. AHLs also exert biological effects on human cells and influence oral diseases such as periodontitis and oral squamous carcinoma. Researchers developed AHL inhibitors to interfere with the quorum sensing process and interrupt the communication between bacteria. These inhibitors can be classified into three main categories based on their mechanisms of action to AHLs: AHL synthesis disruptors, AHL competitive inhibitors and AHL enzymatic degraders. These AHL inhibitors can be important tools in the fight against bacterial infections, particularly those caused by Gram-negative bacteria.

**Conclusion:**

The literatures indicate that AHLs, as quorum sensing molecules, influence bacterial communication. AHLs have a significant impact in bacterial pathogencity and play a potential role in the pathogenesis of oral diseases. Researchers have developed AHL inhibitors to disrupt bacterial quorum sensing, preventing bacteria from forming biofilms or expressing virulence factors. These studies on AHLs represent a new research direction to develop novel therapeutic strategies to manage oral diseases.

## 1 Introduction

The oral cavity is a micro-community and micro-ecosystem within the human body. Over 700 species of microorganisms inhabit the oral cavity, including the surfaces of teeth, the tongue, the oral mucosa, the hard palate, and the gingival crevicular fluid. These microorganisms form biofilms, which are communities of microbes enmeshed in a self-produced matrix of polymeric substances. Biofilms provide a protective environment that enhances microbial survival and facilitates interactions among different species, leading to the development of a highly organized and spatially aware community. This complex community is more than just a collection of individual species; it functions as a unified system. Within this context, Gram-negative bacteria are important oral pathogenic bacteria associated with systemic inflammation. Initially existing in a free-floating, planktonic state, these bacteria can develop into diverse, mature subgingival plaques (1). This transformation is regulated by the quorum sensing (QS) system, a communication network that allows bacteria within this dynamic microbial community to coordinate their behaviours.

**Figure 1:**
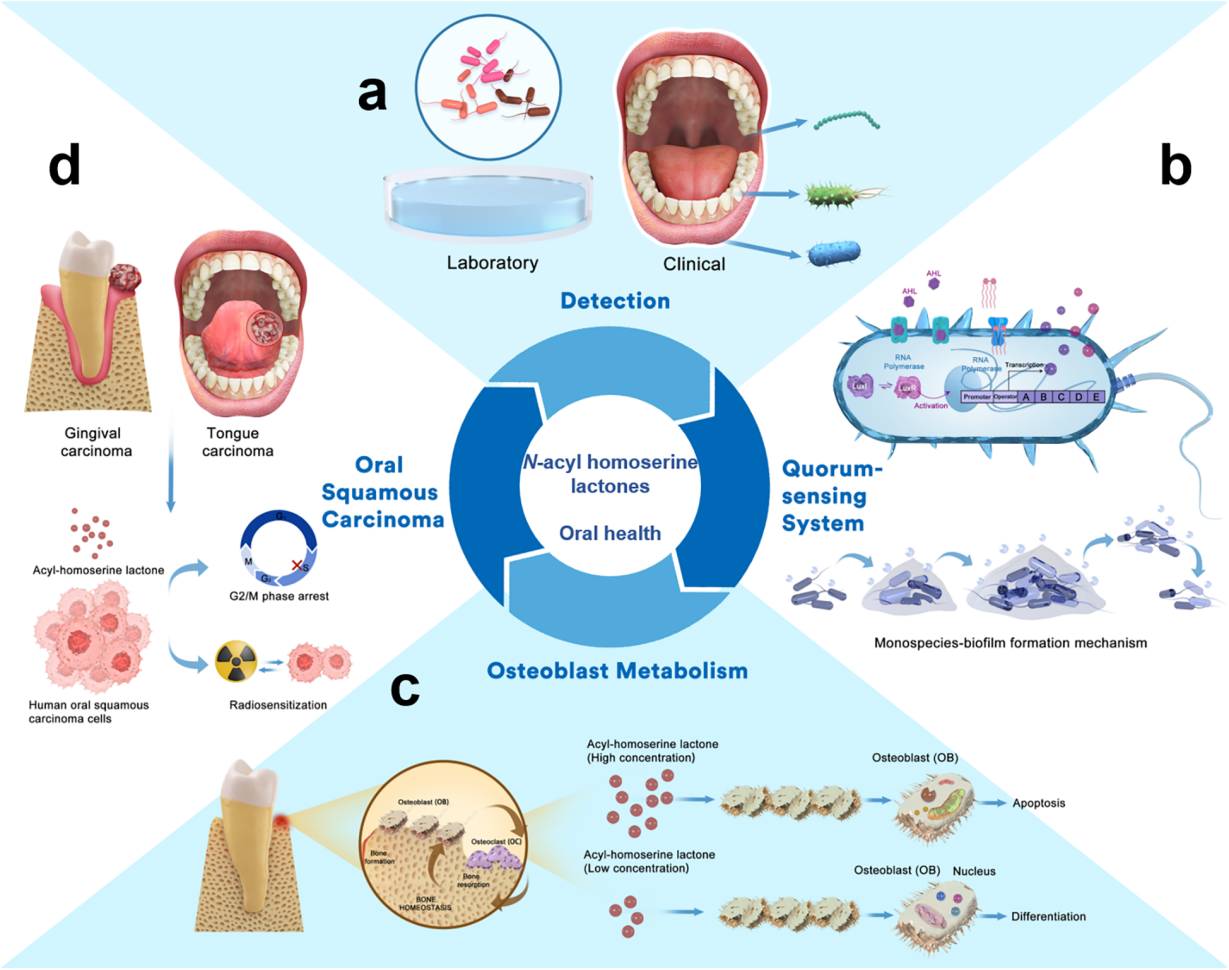
*N*-acyl homoserine lactones and oral health. **(a)** AHLs detected from laboratory and clinical samples. **(b)** AHLs regulate the QS system in oral bacteria and affect the formation of biofilms. **(c)** AHLs regulate osteoblast metabolism and the inner biological process. **(d)** AHLs exhibit antiproliferative effects against human oral squamous carcinoma cells.

There are various QS signalling molecules, known as autoinducers (AIs). Gram-positive bacteria use mainly autoinducer peptides (AIP). Gram-negative bacteria use *N*-acyl homoserine lactones (AHLs) which are among the most intensively studied family of AIs. AHLs are critical intraspecies signalling molecules that significantly influence host biological behaviours (2, 3). Bacteria activate AHL-related intraspecific signal transmission while abiding by specific regulations based on the QS system. This system consists of two components, LuxI-type and LuxR-type proteins. The LuxI-type proteins are AHL synthases that catalyze the synthesis of AHLs, while LuxR-type proteins are transcription factors responsible for the perception of AHLs. The binding of AHLs to LuxR results in the stabilization and dimerization of LuxR (4). When AHLs reach a threshold relative to the microbial population density, the LuxR-AHL complex binds to a conserved 20-bp palindrome termed “*lux box*” and then activates the expression of target genes. These genes regulate specific bacterial behaviours, such as bioluminescence, biofilm formation, plasmid conjugation, motility, and aggregation. This allows Gram-negative bacteria to adapt to environmental changes and cell physiological functions (5). LuxI/LuxR are key factors that drive QS in bacteria through secretion and perception of the signalling molecules. Therefore, AHLs are potentially valuable targets for studying oral microbes and diseases (Figure 1).

The chemical structure of AHLs consists of two parts: a homoserine lactone (HSL) ring and a variable acyl side chain. AHLs are generally divided into short-chain AHLs (C_4_–C_8_) and long-chain AHLs (C_10_–C_18_) according to the length of their side chains. Additionally, the variety of AHLs’ structures is caused by differences in the R and substituent groups in the acyl chain. OH- and O- groups, as common substituents, often replace the H atom at the C_3_ position of AHLs. Long-established physicochemical techniques and whole-cell sensing systems are reliable for this purpose (4). Physicochemical techniques mainly include high-performance liquid chromatography with ultraviolet detection (6), gas chromatography with mass spectrometry (7, 8), high-performance liquid chromatography-mass spectrometry (9, 10), ultra-performance liquid chromatography, and nanoliquid chromatography (11). Whole-cell sensing systems (12) and bioluminescence-based whole-cell sensing systems (13) in bacterial culture media (4) have been used to detect long- and short-chains at extremely low analyte levels, even at sub-attomole levels. These systems exhibit high sensitivity and selectivity, and enhance the speed of the analytical process. They are based on genetically engineered bacteria and liquid chromatography tandem mass spectrometry. High sensitivity allows for the detection of AHLs in physiological samples.

Research focusing on AHLs in oral health provides insights into the management of oral health associated with bacterial biofilm and virulence. By elucidating the role of AHLs in interspecies bacterial communication, we can uncover pathogenic mechanisms underlying oral diseases. Biofilms confer resistance to host immune responses and antibiotics by forming a protective barrier, creating unfavourable microenvironments, altering bacterial growth rates and gene expression, and harbouring a small population of dormant “persister” cells that are highly tolerant to antibiotics and can repopulate the biofilm once treatment has ceased. Currently, antimicrobial resistance (AMR) represents a critical global health crisis, driven by the adaptive evolution of bacterial pathogens that nullify the efficacy of conventional antibiotic therapies. Targeting QS systems, specifically by disrupting AHL-mediated signalling, offers a novel strategy to control bacterial infections by preventing their orchestrated pathogenic behaviour without promoting resistance. Thus, by understanding the role of AHLs, new strategies can be developed to inhibit biofilm formation and enhance treatment efficacy. AHLs also influence the expression of genes related to bacterial pathogenicity and virulence. Exploring how these molecules regulate harmful bacterial behaviours provides potential targets for therapeutic intervention, reducing the impact of oral infections. Unlike traditional antibiotics that kill bacteria, AHL inhibitors could limit bacterial virulence without promoting resistance. However, current understanding of AHLs comes from non-oral models. It is needed to identify which AHLs are specifically relevant to oral pathogens and how they influence disease progression in the unique environment of the oral cavity. Bridging basic research on AHLs to practical applications in oral healthcare remains a challenge. Thus, an overview on AHLs in oral health holds promise for advancing our understanding of bacterial communication in oral environment. Addressing these research gaps will be vital in translating theoretical insights into practical applications that improve oral care.

**Figure 2:**
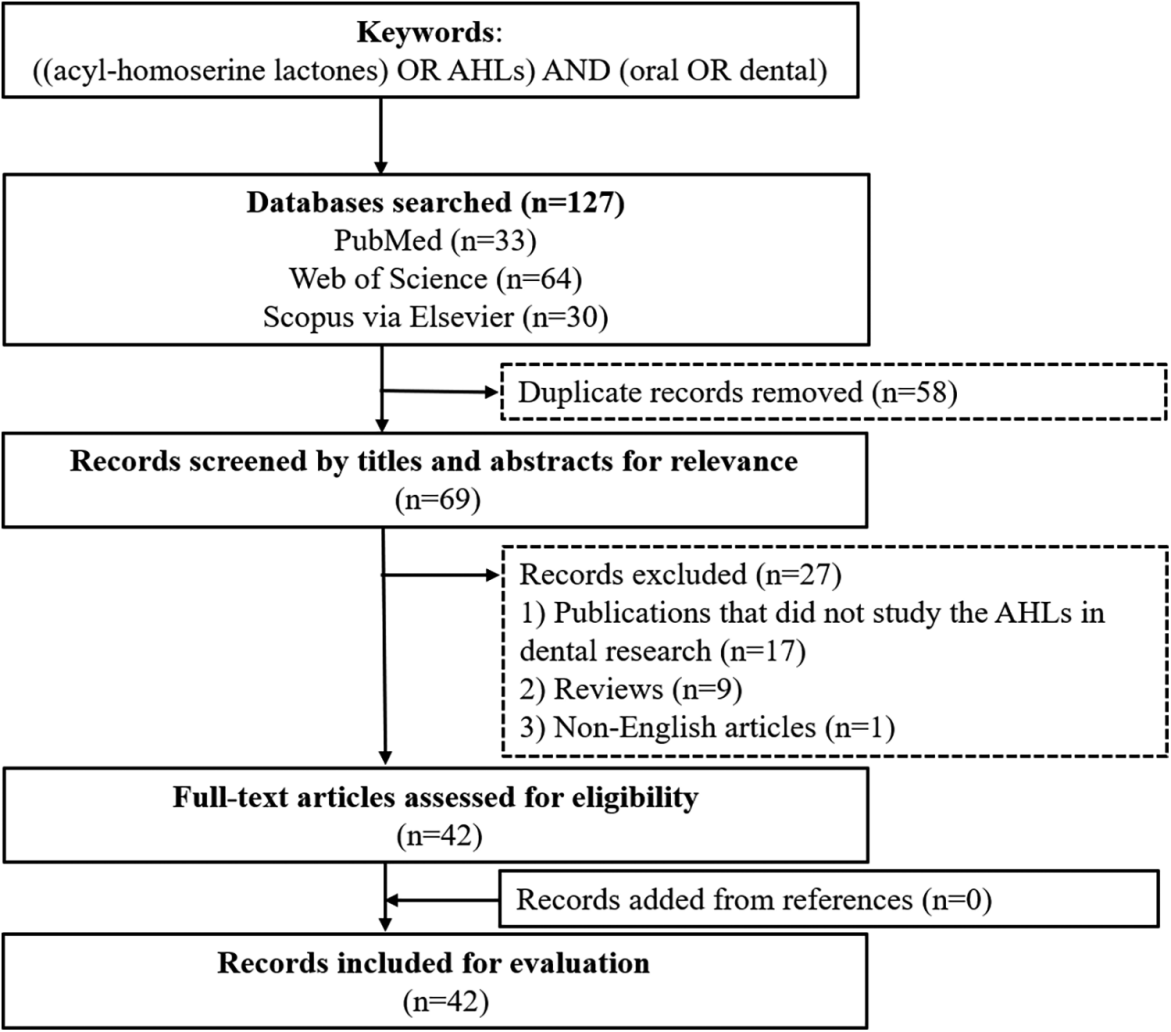
Flowchart of the literature search.

This review involved a systematic search of English language articles in PubMed, Web of Science and Scopus via Elsevier. The keywords used for relevant articles were “[(acyl-homoserine lactones) OR AHLs] AND (oral OR dental)”. Publications published prior to 30 June 2024 were selected. The search yielded 127 potentially relevant publications, and a total of 42 articles were included in this review (Figure 2).

## 2 Studies on AHLs in the oral environment

### 2.1 Detection of AHLs from laboratory and clinical samples

In the early 2000s, researchers first proposed that diffusible small-molecule signals among members of the same micro-community were critical for the regulation of gene responses and plaque maturation. These signals were later determined to correspond to AHLs (1). As a major class of AI signals produced by Proteobacteria, AHLs commonly comprise a homoserine lactone ring carrying an acyl chain of C_4_–C_18_ in length (14). A rather short-chain-length AHL, *N*-octanoyl-L-homoserine lactone (C_8_-HSL), was isolated from a laboratory monospecies sample of the oral bacteria *Porphyromonas gingivalis* (*P. gingivalis*)*.* Meanwhile, multi-species oral biofilms formed by *Streptococcus oralis*, *Veillonella parvula*, *Actinomyces naeslundii*, *Fusobacterium nucleatum*, *Aggregatibacter actinomycetemcomitans* (*A. actinomycetemcomitans*), and *P. gingivalis* cultures contain small amounts of QS signalling *N*-3-oxo-octanoyl-homoserine lactone (OC_8_-HSL) (15).

Several AHLs have been previously described in bacteria clinically isolated from human oral samples and identified using methods such as high-resolution mass spectrometry (16). Bacterial strains, such as *Enterobacter* spp., taken from the human tongue surface were found to produce C_8_-HSL and *N*-dodecanoyl-homoserine lactone (C_12_-HSL) (17, 18). Furthermore, *N*-hexanoyl-L-homoserine lactone (C_6_-HSL), C_8_-HSL, *N*-decanoyl-L-homoserine lactone (C_10_-HSL), and C_12_-HSL were identified from dentin caries (19). Additionally, *N*-butyryl-L-HSL (C_4_-HSL), C_6_-HSL, C_8_-HSL, and *N*-hexadecanoyl-L-homoserine lactone (C_16_-HSL) were identified in bacteria isolated from a dental plaque biofilm sample (20).

The OC_8_-HSL was mostly found in saliva samples from healthy donors (21). It was also isolated from teeth extracted from healthy individuals, patients with dental cavities, and individuals with gastrointestinal disease (4). *N*-tetradecanoyl-L-homoserine lactone (C_14_-HSL) and *N*-octadecanoyl-L-homoserine lactone (C_18_-HSL) have been detected in the saliva of patients with dental caries. C_8_-HSL and occasionally C_14_-HSL have been found in patients with periodontal disease (21) (Table 1). Overall, AHLs' presence in both laboratory and clinical samples underscores their significance as an integral role in the communication and coordinated behaviour of bacterial communities in the oral cavity, suggesting their broad involvement in oral health and disease.

**Table 1:** N-acyl homoserine lactones identified from the bacteria at the different sites in human oral cavity.

*N*-acyl homoserine lactone	Site	Bacteria	Authors, year
C4-HSL *N*-butyryl-L-homoserine lactone	Dental plaque from carious teeth	*Citrobacter amalonaticus*	(20)
C6-HSL *N*-hexanoyl-L-homoserine lactone	Carious dentin from carious teeth	*Burkholderia sp.*	(19)
C8-HSL *N*-octanoyl-L-homoserine lactone	Dental plaque from carious teeth	*Citrobacter amalonaticus*	(20)
	Saliva from people with caries	Not reported	(16, 21)
	Saliva from people with periodontal disease		
	Extracted carious teeth		
	Extracted teeth due to periodontal disease		
	Posterior dorsal surface of tongue from people with no significant oral disease	*Klebsiella pneumoniae*	(17)
		*Pseudomonas putida*	
	Carious dentine	*Burkholderia sp.*	(19)
	Dental plaque from carious teeth	*Citrobacter amalonaticus*	(20)
C10-HSL *N*-decanoyl-L-homoserine lactone	Carious dentine from carious teeth	*Burkholderia sp.*	(19)
C12-HSL *N*-dodecanoyl-L-homoserine lactone	Posterior dorsal surface of tongue from people with no significant oral disease	*Enterobacter sp.*	(18)
		*Klebsiella pneumoniae*	
		*Pseudomonas putida*	(17)
	Carious dentine from carious teeth	*Burkholderia sp.*	(19)
C14-HSL *N*-tetradecanoyl-L-homoserine lactone	Saliva from people with caries	Not reported	(16, 21)
	Saliva from people with periodontal disease		
C16-HSL *N*-hexadecanoyl-L-homoserine lactone	Dental plaque from carious teeth	*Citrobacter amalonaticus*	(20)
C18-HSL *N*- octadecanoyl -L-homoserine lactone	Saliva from people with caries	Not reported	(16, 21)

### 2.2 AHL-regulated QS system in oral bacteria

Given the existence of AHL-mediated QS networks in the oral microbial environment, studies have focused on AHL-related interactions in oral commensal microorganisms (16). Researchers have previously assumed that Gram-negative periodontal organisms lack *N*-acyl HSL-dependent signalling systems (22). To determine whether AHL-related systems exist, one study investigated representative Gram-negative bacteria in the oral environment for a well-known putative third family of homoserine lactone synthetase S (HdtS) by introducing the genomic libraries of *P. gingivalis* W50 DNA into *Escherichia coli* strains containing either the lux-based AHLs reporter pSB401 or pSB1075 (23). These synthases tested negative for each of the transformants required for bioluminescence and the characteristics accompanying satellitism (15). Bioluminescence and satellitism are indicative tests commonly used to identify QS activity, specifically the presence and functionality of AHL synthases. The negative test result suggested that the transformants did not produce or respond to AHLs, implying that *P. gingivalis* likely lacked an AHL-based QS system. Despite the lack of evidence of an AHL-related system in *P. gingivalis*, researchers have investigated the influence of synthetic *N*-acyl HSLs on protein growth and production in *P. gingivalis*. They demonstrated that synthetic AHLs (*N*-tetradecanoyl HSL and acyl-CoA) completely and dose-dependently inhibit the growth of *P. gingivalis* strains and alter their SDS-PAGE expression profiles (24). Thus, *P. gingivalis* possesses a specific receptor that operates as an *N*-acyl HSL transcriptional activator and receives AHLs, complying with a mechanism similar to that of other Gram-negative bacteria (25). *P. gingivalis* W8326 was later found to contain an open reading frame with a 25% identity and 48% amino acid similarity to HdtS, indicating the presence of at least one putative AHL synthase in *P. gingivalis* W8326 (24).

Two other critical proteins were observed: LuxI, which catalyses the acylation and lactonisation reactions between the substrates *S*-adenosylmethionine and hexanoyl-ACP and then synthesises AHLs, and LuxR, which is the cytoplasmic receptor for AHLs and a transcriptional activator of the virulence-related operon (14). *P. gingivalis* was found to contain a LuxR homologue, Community Development and Hemin Regulator, which controls the transcription of the *hmu* operon responsible for iron/hemin uptake (26, 27). Whole-genome sequencing analysis showed that *Citrobacter amalonaticus* (*C. amalonaticus*) possesses the QS signalling synthase gene of a LuxI/LuxR functional pair clustered on the same chromosome.

Major Gram-negative periodontal pathogens, such as *P. gingivalis* and *A. actinomycetemcomitans*, utilise AHLs to regulate dental biofilm development. Gram-positive oral bacteria cannot produce AHLs but possess LuxR homologues, known as LuxR orphans, which can interact with QS molecules produced by other microorganisms in the environment (28). For example, a gene that belongs to the LuxR family of regulatory proteins has been predicted in the genome of *Streptococcus mutans* (29). *Staphylococcus aureus* can respond to OC_12_-HSL produced by *Pseudomonas aeruginosa* (*P. aeruginosa*) in a saturable and specific manner, thus inhibiting the production of exotoxins and enhancing the expression of protein A, an important surface protein involved in several virulence mechanisms (30).

Even though earlier assumptions suggested a lack of AHL-dependent signalling in certain oral bacteria like *P. gingivalis*, recent studies have demonstrated the presence of AHL receptors and potential synthase genes. AHLs differentially regulate subsequent bacterial communication to express specific gene sets, ultimately triggering behavioural changes (15, 24). Evidently, the AHL-regulated QS system plays a crucial role in the coordination of behaviours among oral bacteria, influencing biofilm formation and pathogenicity. This process can aggravate oral biofilm-related diseases.

### 2.3 Effect of AHLs on oral diseases

In addition to being integral components of bacterial QS systems, AHLs exert biological effects on human cells and influence oral diseases (Table 2). *N*-(3-oxododecanoyl)-L-homoserine lactone (OdDHL, OC12-HSL) is secreted by *P. aeruginosa*, a Gram-negative opportunistic pathogen detected in periapical disease and periodontitis. OdDHL helps construct a niche suitable for *P. aeruginosa* invasion and reproduction, further aggravating bone destruction. OdDHL can regulate osteoblast metabolism (apoptosis and differentiation) by mediating intracellular calcium [(Ca2^+^)_i_] ﬂuctuations and spatial correlation in preosteoblastic MC3T3-E1 cells. Different concentrations of OdDHL trigger opposing osteoblast fates. At 50 μM, OdDHL inhibits osteoblast differentiation by promoting mitochondrial-dependent apoptosis and negatively regulating osteogenic marker genes, including Runx2, Osterix, bone sialoprotein, and osteocalcin. Additionally, it elevated Ca2^+^_i_ in spatially autocorrelated osteoblasts. At 30 μm, OdDHL promoted osteoblast differentiation and apoptosis. Thus, AHLs affect osteoblast metabolism, the most important biological process for maintaining bone mass (35). Moreover, OdDHL plays a positive role in antitumor activity (36). Structure-activity relationship studies have found that C_12_-HSL exhibits antiproliferative effects against human oral squamous carcinoma cells derived from gingival carcinoma (Ca9-22 cells) and tongue cancer (human tongue squamous carcinoma cells) as well as radiation-sensitising effects against Ca9-22 cells (34). AHLs not only modulate bacterial QS but also exert profound effects on human host cells, influencing processes critical to oral health such as osteoblast metabolism and bone homeostasis. This regulation of osteoblast activity highlights its potential impact on bone integrity in oral diseases like periodontitis. Additionally, the antiproliferative and radiation-sensitizing effects on oral squamous carcinoma cells suggest therapeutic avenues for cancer treatment. Generally, these findings underscore the multifaceted role of AHLs in oral health and disease.

**Table 2:** Laboratory studies on *N*-acyl homoserine lactones and their analogues.

*N*-acyl homoserine lactone (analogue)	Main findings	Authors, year
C6-HSL analogues *N*-hexanoyl-L-homoserine lactone analogues	C6-HSL analogues inhibited *Porphyromonas gingivalis* bioﬁlm formation	(31)
C6-HSL *N*-hexanoyl-L-homoserine lactone	C6-HSL altered periodontal bacterial composition profile and increased in relative presence of *Prevotella sp*. and *Peptostreptococcus sp*.	(16, 21)
C12-HSL analogues *N*-dodecanoyl-L-homoserine lactone analogues	C12-HSL analogues inhibited *Porphyromonas gingivalis* growth and bioﬁlm formation.	(31)
	C12-HSL analogues abolished lactic acid accumulation by the biofilms without affecting biofilm growth and reduced the relative abundance of *Streptococcus spp.* in favor of *Veillonella spp.*	(32)
	C12-HSL analogues induced endoreduplication, inhibited proliferation and promoted radiation sensitivity on human oral squamous carcinoma cells.	(33, 34)
OC12-HSL *N*-(3-oxododecanoyl)-homoserine lactone	OC12-HSL at high concentration inhibited osteoblast differentiation by promoting mitochondrial-dependent apoptosis, whereas at low concentration promoted osteoblast differentiation concomitantly with cell apoptosis.	(35, 36)
C14-HSL *N*-tetradecanoyl-L-homoserine lactone	C14-HSL altered protein production and inhibited growth of *Porphyromonas gingivalis*.	(24)

## 3 AHL inhibitors

Given the critical role of AHLs in the QS system, an increasing number of studies utilized the AHL inhibitors to disrupt the QS processes in bacteria. According to their mechanisms of action and structural similarities to AHLs, three primary types of AHL inhibitors are included: AHL synthesis disruptors, AHL competitive inhibitors and AHL enzymatic degraders.

### 3.1 AHL synthesis disruptors

AHL synthesis inhibitors disrupt the production of signalling molecules. Some natural products inhibit AHLs' biosynthesis, such as halogenated furanones from the marine alga *Delisea pulchra* (37). New bacterial species related to marine organisms were isolated and characterised as potential candidates for antimicrobial and antibiofilm treatments. Through AHL-based QS, bromoageliferin and oroidin, which are produced by marine organisms, trigger biofilm detachment in Gram-negative bacteria (38, 39).

A variety of chemical compounds have been investigated for their quorum quenching functions such as 4-nitro-pyridine-N-oxide from garlic cloves (40). However, these compounds have toxic and carcinogenic effects and offer poor stability in aqueous solutions. These factors restrict their use as antimicrobials (41). Macrolide antibiotics have been found to modulate AHLs' biosynthesis at non-lethal concentrations, though their precise mechanism of inhibition remains unclear (42). Additionally, pyrimidinone compounds have been explored as potential antagonists of AHLs, with a patent applied for their use in treating periodontal disease in the oral cavity (43). Pyrimidinones effectively inhibit C_6_-HSL and C_8_-HSL synthesis, toxin production by *Burkholderia glumae*, and biofilm formation by *P. aeruginosa* and *P. gingivalis*.

### 3.2 AHL competitive inhibitors

Analogues of AHLs mimic the structure of natural AHLs and competitively bind to the QS receptors, preventing the actual AHLs from activating the signalling pathway. Treatment with synthetic *N*-acyl HSL analogues inhibits biofilm formation by *P. gingivalis*, a primary etiological agent of periodontal disease. Treatment with 100 µM C_6_-HSL and C_12_-HSL analogues remarkably decreased *P. gingivalis* biofilm formation in a quantitative and structural (three-dimensional) manner, without inducing quantitative or qualitative differences in primary cell attachment. This suggests that the analogues affect the developmental stages of microcolonies and biofilm formation of *P. gingivalis* (31). However, this type of AHL inhibitors does not affect initial attachment or mature biofilms. The molecule 3-oxo-N-(2-oxocyclohexyl)-dodecanamide (3-oxo-N), a structural homologue of C_12_-HSL, altered the ecological homeostasis of *in vitro* dental plaques by reducing the cariogenic potential through minimisation of lactic acid accumulation. However, it did not notably inhibit biofilm formation. Thus, 3-oxo-N is a potential compound for maintaining a healthy, non-cariogenic ecology in *in vivo* dental plaque. In addition to preventing caries, 3-oxo-N significantly alters species composition and ameliorates an unhealthy ecology by suppressing the maturation of *Megasphaera micronuciformis* and *Solobacterium moorei*, which were previously found in periodontal sites (32). The combined application of *N*-acyl HSL analogues and typical antibiotics reduces the viability of *P. gingivalis* cells in biofilms. A C_6_-HSL analogue at a final concentration of 100 µmol L^−1^ combined with 1 µg ml^−1^ minocycline or cefuroxime exerted strong inhibitory effects on biofilm formation. This analogue reduced the thickness, volume, and matrix production of the biofilm, allowing easy antibiotic penetration into *P. gingivalis* colonies and demonstrating higher efficacy than a single application of antibiotics or analogues (25). Additionally, S-allyl-cysteine from garlic extracts, with a structure similar to that of AHLs, yielded excellent results in the treatment of a multi-species bacterial infection when combined with an antibiotic and/or antiseptic. This is especially effective for dental infections, including periodontal diseases such as gingivitis, periodontitis, pericoronitis, peri-implantitis, and related inflammation (44). AHL analogues of reduced the required antibiotic concentrations and treatment durations for controlling periodontal biofilm growth. This finding suggests a novel therapy for the treatment of periodontal diseases without the adverse effects of antibiotics or bacterial tolerance to antibiotics.

A previous study evaluated and improved the cytotoxicity of acridine-based AHL analogues in radiosensitizing human oral squamous carcinoma cells and human tongue squamous carcinoma cells. AHL analogues induce G2/M phase arrest and polyploidy. Synergised with *X*-irradiation, AHL analogues also inhibit the clonogenic survival of human tongue squamous carcinoma cells, which is related to mitotic failure following enhanced expression of Aurora A and B. Active AHL analogues can suppress the growth of human tongue squamous carcinoma cells and promote radiosensitisation (33). Thus, AHL analogues are considered new druggable targets and effective chemotherapeutic medicines. They can be used in treating human oral squamous carcinoma cells and in conjunction with radiotherapy as a postoperative treatment for long-term control.

AHL analogues show significant potential as innovative therapeutic agents for controlling bacterial biofilm formation, enhancing antibiotic efficacy, and serving as chemotherapeutic agents, particularly in the treatment of periodontal diseases and oral squamous carcinoma. Further research is necessary to fully understand their benefits and optimize their application in clinical settings.

### 3.3 AHL enzymatic degraders

AHL enzymatic degraders break down AHLs. They consist of three main types: lactonases, which target the lactone bond; acylases, which target the amide linkage; and oxidoreductases, which target the acyl chain. Gram-positive bacterial and eukaryotic (e.g., plant) cells produce enzymes such as lactonases and acylases that break down AHLs (45). Among these enzymes, lactonases are the most widely characterised and have been the focus of recent research. AHL lactonases hydrolytically cleave the ester bond of the HSL ring to form a homologous AHL derivative. The AHL lactonase Aii20j was effective against multi-species biofilms formed by several oral pathogens. It also significantly inhibited biofilm formation in *in vitro* oral biofilm models inoculated with saliva samples from healthy individuals and patients with oral diseases (21). Another AHL lactonase, est816, was shown to inhibit *A. actinomycetemcomitans* biofilm formation and virulence release, resulting in anti-inflammatory effects and smoothing periodontitis in rats (46). These findings demonstrate the potential of AHL enzymatic degraders as a promising biocontrol strategy to mitigate antimicrobial resistance. These enzymes—particularly lactonases—are capable of impairing the signaling pathways essential for biofilm formation and virulence expression. The demonstrated effectiveness of lactonases, such as Aii20j and est816, in inhibiting biofilm formation and reducing pathogenicity in both single-species and multi-species settings highlights their relevance in treating oral diseases.

Overall, by targeting bacterial communication specifically, these AHL inhibitors offer a precise approach to controlling bacterial infections without fostering resistant phenotypes, positioning them as innovative tools in combating microbial resistance and enhancing dental health.

## 4 Limitations in the current knowledge of AHLs in oral health

### 4.1 Methods of detecting AHLs in oral samples

Although existing analytical methods are most commonly used for detecting AHLs in biological and clinical samples, quantifying a small number of AHLs in culture media remains challenging due to several limitations. Hence, it is difficult to verify and quantify AHLs in oral samples and monospecies culture supernatants. A study in 2001 indicated that no *A. actinomycetemcomitans* strain could produce the tested AHLs (22). Previous reporters were primarily based on *P. aeruginosa* and *Vibrio* spp., which are structurally distinct from those found in oral bacteria. Moreover, only a few oral species have been tested, and they are not wholly representative of the complex multi-species biofilms in the oral cavity. Thus, identifying specific species in the AHL-mediated QS system is both challenging and important (47).

### 4.2 Physiological mechanisms regulated by AHLs in oral health

A thorough schematic representation of the AHL-related QS pathway in common oral bacteria remains unknown. Previous studies have mainly focused on the association between AHLs and *P. gingivalis*. Evidence has shown that the HdtS-CdhR system in *P. gingivalis* is similar to the LuxI-LuxR system. Also, C_8_-HSL was detected in *P. gingivalis* laboratory samples. Thus, researchers proposed the following hypothesis: AHL-regulated QS system inherently controls the expression of specific virulence factors in *P. gingivalis*. These factors are implicated in the aetiology of human periodontal disease due to the virulence associated with the elaboration of cysteine proteases, Arg-gingipain (Rgp), and Lys-gingipain (Kgp) (2, 3). Whether AHLs affect the biomechanism of the expression of these main virulence genes through the QS system should be explored.

In addition to *P. gingivalis*, specific AHLs produced by other major Gram-negative oral bacteria in the biofilm matrix were not detected. These AHLs are also critical, as cognate AHLs can activate QS circuits, and non-cognate AHLs can inhibit the same receptor with similar structures (16). Therefore, future studies should conduct separate qualitative detections of various oral pathogens and consider the complexity of oral microorganisms when building oral biofilm models.

Research has demonstrated that AHLs indirectly inhibit immunosuppressive cytokines and chemokines and trigger the immunostimulating ones (48), by regulating the biological behaviours of oral pathogens. Thses pathogens use cell envelopes and exoproduct virulence to attach to the surface, continuously degrading host cell tissues and immune effector molecules and ultimately invading epithelial cells (49–51). No evidence has been found regarding the relationship between AHLs and immune-related inflammatory diseases extending into the oral environment. Monitoring the dynamic process of AHLs within cells is challenging. Varying concentrations of AHLs in immunocytes results in contrasting effects. Therefore, investigating the role of AHLs in either suppressing or stimulating cytokines and chemokines in oral inflammatory diseases is necessary. Understanding the correlation between AHLs and inflammatory mediators is crucial for further developing strategies involving AHL analogues or antagonists.

## 5 Discussion

The authors chose PubMed, Web of Science, and Scopus for researching AHLs in oral health for the following reasons. First, comprehensive coverage: these databases provide a wide-ranging collection of scholarly articles across various disciplines, including medicine, biology, and dentistry, ensuring comprehensive coverage of the topic. Second, the inclusion of reputable sources: all three databases are well-regarded for indexing peer-reviewed journals, ensuring that the information is credible and of high scientific quality. Third, interdisciplinary integration: since AHLs impact both microbiology and oral science, these databases collectively cover the necessary interdisciplinary scope, facilitating access to relevant studies from multiple scientific domains.

Given the limitations and drawbacks of conventional methods, rigorous studies on fast-responsive methods for detecting AHLs are needed to accelerate the entire analytical process for biological samples. While AHLs can be extracted and identified from mono-cultured strains found in typical human oral samples such as carious dentine, dental plaque, and the tongue surface, there remains a significant concern regarding the comparatively low prevalence of these reported bacteria (*C. amalonaticus*, *Burkholderia* sp., *Klebsiella pneumoniae*, *Pseudomonas putida*, and *Enterobacter* sp.) in the human oral cavity. The oral cavity serves as the entry portal for clinically relevant environmental bacteria from external sources to colonise the human mucosa or tooth surface and enter the gastrointestinal tract. Evidence highlighting the causation between intestinal Gram-negative bacteria, or their specific AHLs, and oral diseases (dental caries, periodontitis, and oral cancer) is lacking. Hence, further investigation is needed on the regularly reported bacteria at the environment-oral cavity interface and their role as vectors potentially involved in virulence, AHLs' production, and transmission. Additionally, researchers should consider interfering molecules related to the complexity of biological matrices, such as bacterial interactions. This strategy influences the signalling transmission pathways and indirectly affects the concentration of AHLs at different sites in the oral cavity. Further studies are necessary to confirm the precise types and quantities of AHLs in multi-species compositions.

Further investigation of the role of AHLs in mediating the participation of Gram-negative bacteria in the development of oral diseases is needed. The major goal is to understand cell-to-cell communication processes at the molecular, cellular, and population levels regarding the amount and/or type of specific oral pathologies (14). We should elucidate the evolution of AHLs in bacterial populations, the expression of virulence factors, and the regulatory strategies. This understanding will enable the development of biotechnological therapies to manipulate bacterial behaviour. AHLs also serve as potential biomarkers for the diagnosis and management of several oral diseases (4). Methods for detecting AHLs can be used for the early diagnosis and monitoring of bacterium-associated infectious oral diseases, especially those caused by antibiotic-resistant bacteria.

Recent advances in the development of novel AHL inhibitors have raised the possibility of their application in the prevention and treatment of oral infectious diseases. Oral biofilms remain a significant target in the development of chronic infectious oral diseases. Biofilms have lower antibiotic susceptibility than planktonic bacteria and resist thorough elimination either by chemical control agents or mechanical debridement (45). Traditional antibiotics face various problems, such as gastrointestinal toxicity, microflora imbalance, and drug resistance (52). Controlling antibiotic abuse and developing new drugs to inhibit plaque biofilm formation are primary research goals. AHL inhibitors can interfere with the communication within and among oral pathogens. They prevent initial microbial attachment or penetration of the biofilm matrix and reduce the associated cells. As a result, they have become a new strategy due to their unique functions and effects (53).

## 6 Conclusion

Studies have demonstrated the characteristics of AHLs and their biological effects on oral bacterial behaviour and the regulation of oral cells. Innovations in this novel QS-based strategy can be applied to inhibit plaque biofilm formation, reduce bacterial toxicity, and help control host inflammatory responses and biomechanisms. These studies represent a new direction for microbiology and pathology research in dentistry and offer a promising avenue for reducing AMR by impeding bacterial communication and cooperation necessary for virulence, without exerting selective pressure for resistance. Based on these findings, further investigation is required to explore other AHLs and develop novel therapeutic strategies to prevent and manage oral diseases.

## References

[1] Kolenbrander PE Palmer RJ Rickard AH Jakubovics NS Chalmers NI Diaz PI. Bacterial interactions and successions during plaque development. Periodontol 2000. (2006) 42:47–79. 10.1111/j.1600-0757.2006.00187.x 16930306

[2] Williams P Winzer K Chan WC Cámara M. Look who’s talking: communication and quorum sensing in the bacterial world. Philos Trans R Soc Lond B Biol Sci. (2007) 362(1483):1119–34. 10.1098/rstb.2007.2039 17360280 PMC2435577

[3] Withers H Swift S Williams P. Quorum sensing as an integral component of gene regulatory networks in gram-negative bacteria. Curr Opin Microbiol. (2001) 4(2):186–93. 10.1016/S1369-5274(00)00187-9 11282475

[4] Kumari A Pasini P Daunert S. Detection of bacterial quorum sensing N-acyl homoserine lactones in clinical samples. Anal Bioanal Chem. (2008) 391(5):1619–27. 10.1007/s00216-008-2002-3 18408921

[5] Passador L Cook JM Gambello MJ Rust L Iglewski BH. Expression of *Pseudomonas aeruginosa* virulence genes requires cell-to-cell communication. Science. (1993) 260(5111):1127–30. 10.1126/science.8493556 8493556

[6] Reimmann C Beyeler M Latifi A Winteler H Foglino M Lazdunski A The global activator GacA of *Pseudomonas aeruginosa* PAO positively controls the production of the autoinducer N-butyryl-homoserine lactone and the formation of the virulence factors pyocyanin, cyanide, and lipase. Mol Microbiol. (1997) 24(2):309–19. 10.1046/j.1365-2958.1997.3291701.x 9159518

[7] Charlton TS de Nys R Netting A Kumar N Hentzer M Givskov M A novel and sensitive method for the quantification of N-3-oxoacyl homoserine lactones using gas chromatography-mass spectrometry: application to a model bacterial biofilm. Environ Microbiol. (2000) 2(5):530–41. 10.1046/j.1462-2920.2000.00136.x 11233161

[8] Cataldi TRI Bianco G Palazzo L Quaranta V. Occurrence of N-acyl-L-homoserine lactones in extracts of some gram-negative bacteria evaluated by gas chromatography-mass spectrometry. Anal Biochem. (2007) 361(2):226–35. 10.1016/j.ab.2006.11.037 17207763

[9] Zhang L Murphy PJ Kerr A Tate ME. Agrobacterium conjugation and gene regulation by N-acyl-L-homoserine lactones. Nature. (1993) 362(6419):446–48. 10.1038/362446a0 8464475

[10] Lithgow JK Wilkinson A Hardman A Rodelas B Wisniewski-Dyé F Williams P The regulatory locus cinRI in *Rhizobium Leguminosarum* controls a network of quorum-sensing loci. Mol Microbiol. (2000) 37(1):81–97. 10.1046/j.1365-2958.2000.01960.x 10931307

[11] Fekete A Frommberger M Rothballer M Li X Englmann M Fekete J Identification of bacterial N-acylhomoserine lactones (AHLs) with a combination of ultra-performance liquid chromatography (UPLC), ultra-high-resolution mass spectrometry, and *in situ* biosensors. Anal Bioanal Chem. (2007) 387(2):455–67. 10.1007/s00216-006-0970-8 17165024

[12] De Kievit TR Gillis R Marx S Brown C Iglewski BH. Quorum-Sensing genes in *Pseudomonas aeruginosa* biofilms: their role and expression patterns. Appl Environ Microbiol. (2001) 67(4):1865–73. 10.1128/AEM.67.4.1865-1873.2001 11282644 PMC92808

[13] Swift S Winson MK Chan PF Bainton NJ Birdsall M Reeves PJ A novel strategy for the isolation of luxI homologues: evidence for the widespread distribution of a LuxR:luxI superfamily in enteric bacteria. Mol Microbiol. (1993) 10(3):511–20. 10.1111/j.1365-2958.1993.tb00923.x 7968529

[14] Ng W-L Bassler BL. Bacterial quorum-sensing network architectures. Annu Rev Genet. (2009) 43:197–222. 10.1146/annurev-genet-102108-134304 19686078 PMC4313539

[15] Burgess NA Kirke DF Williams P Winzer K Hardie KR Meyers NL LuxS-Dependent quorum sensing in *Porphyromonas Gingivalis* modulates protease and haemagglutinin activities but is not essential for virulence. Microbiology (Reading). (2002) 148(Pt 3):763–72. 10.1099/00221287-148-3-763 11882711

[16] Muras A Mayer C Otero-Casal P Exterkate RAM Brandt BW Crielaard W Short-chain N-acylhomoserine lactone quorum-sensing molecules promote periodontal pathogens in *in vitro* oral biofilms. Appl Environ Microbiol. (2020) 86(3):e01941–19. 10.1128/AEM.01941-19 31757829 PMC6974637

[17] Chen J-W Chin S Tee KK Yin W-F Choo YM Chan K-G. N-Acyl homoserine lactone-producing *Pseudomonas Putida* strain T2-2 from human tongue surface. Sensors (Basel). (2013) 13(10):13192–203. 10.3390/s131013192 24084113 PMC3859058

[18] Yin W-F Purmal K Chin S Chan X-Y Koh C-L Sam C-K N-Acyl homoserine lactone production by *Klebsiella Pneumoniae* isolated from human tongue surface. Sensors (Basel). (2012) 12(3):3472–83. 10.3390/s120303472 22737019 PMC3376583

[19] Goh SY Tan W-S Khan SA Chew HP Kasim NHA Yin W-F Unusual multiple production of N-acylhomoserine lactones a by *Burkholderia* Sp. Strain C10B isolated from dentine caries. Sensors (Basel). (2014) 14(5):8940–49. 10.3390/s140508940 24854358 PMC4063041

[20] Goh S-Y Khan SA Tee KK Kasim NHA Yin W-F Chan K-G. Quorum sensing activity of *Citrobacter amalonaticus* L8A, a bacterium isolated from dental plaque. Sci Rep. (2016) 6:20702. 10.1038/srep20702 26860259 PMC4748228

[21] Muras A Otero-Casal P Blanc V Otero A. Acyl homoserine lactone-mediated quorum sensing in the oral cavity: a paradigm revisited. Sci Rep. (2020) 10(1):9800. 10.1038/s41598-020-66704-4 32555242 PMC7300016

[22] Frias J Olle E Alsina M. Periodontal pathogens produce quorum sensing signal molecules. Infect Immun. (2001) 69(5):3431–34. 10.1128/IAI.69.5.3431-3434.2001 11292769 PMC98305

[23] Laue BE Jiang Y Chhabra SR Jacob S Stewart GSAB Hardman A The biocontrol strain Pseudomonas Fluorescens F113 produces the rhizobium small bacteriocin, N-(3-hydroxy-7-cis-tetradecenoyl)Homoserine lactone, via HdtS, a putative novel N-acylhomoserine lactone synthase. Microbiology (Reading). (2000) 146(Pt 10):2469–80. 10.1099/00221287-146-10-2469 11021923

[24] Ito A Ito T Yamanaka A Okuda K Yamada S Kato T. N-tetradecanoyl homoserine lactone, signaling compound for quorum sensing, inhibits *Porphyromonas gingivalis* growth. Res J Microbiol. (2010) 1:353–9. 10.3923/jm.2006.353.359

[25] Asahi Y Noiri Y Igarashi J Suga H Azakami H Ebisu S. Synergistic effects of antibiotics and an N-acyl homoserine lactone analog on Porphyromonas Gingivalis biofilms. Journal of Applied Microbiology. (2012) 112(2):404–11. 10.1111/j.1365-2672.2011.05194.x 22093286

[26] Wu J Lin X Xie H. Regulation of hemin binding proteins by a novel transcriptional activator in *Porphyromonas gingivalis*. J Bacteriol. (2009) 191(1):115–22. 10.1128/JB.00841-08 18931136 PMC2612430

[27] Chawla A Hirano T Bainbridge BW Demuth DR Xie H Lamont RJ. Community signalling between *Streptococcus gordonii* and *Porphyromonas gingivalis* is controlled by the transcriptional regulator CdhR. Mol Microbiol. (2010) 78(6):1510–22. 10.1111/j.1365-2958.2010.07420.x 21143321 PMC3017474

[28] Patankar AV González JE. An orphan LuxR homolog of *Sinorhizobium meliloti* affects stress adaptation and competition for nodulation. Appl Environ Microbiol. (2009) 75(4):946–55. 10.1128/AEM.01692-08 19088317 PMC2643561

[29] Wen ZT Nguyen AH Bitoun JP Abranches J Baker HV Burne RA. Transcriptome analysis of LuxS-deficient *Streptococcus mutans* grown in biofilms. Mol Oral Microbiol. (2011) 26(1):2–18. 10.1111/j.2041-1014.2010.00581.x 21214869 PMC3105442

[30] Qazi S Middleton B Muharram SH Cockayne A Hill P O’Shea P N-Acylhomoserine lactones antagonize virulence gene expression and quorum sensing in *Staphylococcus aureus*. Infect Immun. (2006) 74(2):910–19. 10.1128/IAI.74.2.910-919.2006 16428734 PMC1360299

[31] Asahi Y Noiri Y Igarashi J Asai H Suga H Ebisu S. Effects of N-acyl homoserine lactone analogues on *Porphyromonas gingivalis* biofilm formation. J Periodontal Res. (2010) 45(2):255–61. 10.1111/j.1600-0765.2009.01228.x 19778324

[32] Janus MM Crielaard W Zaura E Keijser BJ Brandt BW Krom BP. A novel compound to maintain a healthy oral plaque ecology *in vitro*. J Oral Microbiol. (2016) 8:32513. 10.3402/jom.v8.32513 27476444 PMC4967710

[33] Chai H Hazawa M Hosokawa Y Igarashi J Suga H Kashiwakura I. Novel acridine-based N-acyl-homoserine lactone analogs induce endoreduplication in the human oral squamous carcinoma cell line SAS. Biol Pharm Bull. (2012) 35(8):1257–63. 10.1248/bpb.b12-00033 22863922

[34] Chai H Hazawa M Shirai N Igarashi J Takahashi K Hosokawa Y Functional properties of synthetic N-acyl-L-homoserine lactone analogs of quorum-sensing gram-negative bacteria on the growth of human oral squamous carcinoma cells. Invest New Drugs. (2012) 30(1):157–63. 10.1007/s10637-010-9544-x 20878204

[35] Guo J Wang Z Weng Y Yuan H Yoshida K Ikegame M N-(3-Oxododecanoyl)-homoserine lactone regulates osteoblast apoptosis and differentiation by mediating intracellular calcium. Cell Signal. (2020) 75:109740. 10.1016/j.cellsig.2020.109740 32818672

[36] Guo J Yoshida K Ikegame M Okamura H. Quorum sensing molecule N-(3-oxododecanoyl)-l-homoserine lactone: an all-rounder in mammalian cell modification. J Oral Biosci. (2020) 62(1):16–29. 10.1016/j.job.2020.01.001 31982630

[37] Hentzer M Riedel K Rasmussen TB Heydorn A Andersen JB Parsek MR Inhibition of quorum sensing in *Pseudomonas aeruginosa* biofilm bacteria by a halogenated furanone compound. Microbiology (Reading). (2002) 148(Pt 1):87–102. 10.1099/00221287-148-1-87 11782502

[38] Huigens RW Ma L Gambino C Moeller PDR Basso A Cavanagh J Control of bacterial biofilms with marine alkaloid derivatives. Mol BioSyst. (2008) 4(6):614–21. 10.1039/b719989a 18493660

[39] Huigens RW Richards JJ Parise G Eric Ballard T Zeng W Deora R Inhibition of *Pseudomonas aeruginosa* biofilm formation with bromoageliferin analogues. J Am Chem Soc. (2007) 129(22):6966–67. 10.1021/ja069017t 17500516

[40] Rasmussen TB Bjarnsholt T Skindersoe ME Hentzer M Kristoffersen P Köte M Screening for quorum-sensing inhibitors (QSI) by use of a novel genetic system, the QSI selector. J Bacteriol. (2005) 187(5):1799–814. 10.1128/JB.187.5.1799-1814.2005 15716452 PMC1063990

[41] Hentzer M Givskov M. Pharmacological inhibition of quorum sensing for the treatment of chronic bacterial infections. J Clin Invest. (2003) 112(9):1300–7. 10.1172/JCI20074 14597754 PMC228474

[42] Pechère JC. “Patients” interviews and misuse of antibiotics. Clin Infect Dis. (2001) 33 Suppl 3:S170–3. 10.1086/321844 11524715

[43] Romero M Acuña L Otero A. Patents on quorum quenching: interfering with bacterial communication as a strategy to fight infections. Recent Pat Biotechnol. (2012) 6(1):2–12. 10.2174/187220812799789208 22420877

[44] Stanley SR. Antibacterial Combinations Comprising A Garlic extrtact Or S-allyl Cysteine. European Patent No EP2459149A2: European Patent Office. (2012).

[45] Landini P Antoniani D Grant Burgess J Nijland R. Molecular mechanisms of compounds affecting bacterial biofilm formation and dispersal. Appl Microbiol Biotechnol. (2010) 86(3):813–23. 10.1007/s00253-010-2468-8 20165945

[46] Zhao ZZ Wang J Liu X Wang Z Zheng X Li W N-Acyl homoserine lactones lactonase Est816 suppresses biofilm formation and periodontitis in rats mediated by *Aggregatibacter actinomycetemcomitans*. J Oral Microbiol. (2024) 16(1):2301200. 10.1080/20002297.2023.2301200 38193137 PMC10773656

[47] Shao H Demuth DR. Quorum sensing regulation of biofilm growth and gene expression by oral bacteria and periodontal pathogens. Periodontol 2000. (2010) 52(1):53–67. 10.1111/j.1600-0757.2009.00318.x 20017795

[48] Smith KM Bu Y Suga H. Library screening for synthetic agonists and antagonists of a *Pseudomonas aeruginosa* autoinducer. Chem Biol . (2003) 10(6):563–71. 10.1016/S1074-5521(03)00107-8 12837389

[49] Fan X Liang M Wang L Chen R Li H Liu X. Aii810, a novel cold-adapted N-acylhomoserine lactonase discovered in a metagenome, can strongly attenuate *Pseudomonas aeruginosa* virulence factors and biofilm formation. Front Microbiol. (2017) 8:1950. 10.3389/fmicb.2017.01950 29067011 PMC5641347

[50] Lamont RJ Jenkinson HF. Life below the gum line: pathogenic mechanisms of *Porphyromonas gingivalis*. Microbiol Mol Biol Rev. (1998) 62(4):1244–63. 10.1128/MMBR.62.4.1244-1263.1998 9841671 PMC98945

[51] Yoneda M Kuramitsu HK. Genetic evidence for the relationship of *Porphyromonas gingivalis* cysteine protease and hemagglutinin activities. Oral Microbiol Immunol. (1996) 11(3):129–34. 10.1111/j.1399-302X.1996.tb00347.x 8941765

[52] Contaldo M D’Ambrosio F Ferraro GA Di Stasio D Di Palo MP Serpico R Antibiotics in dentistry: a narrative review of the evidence beyond the myth. Int J Environ Res Public Health. (2023) 20(11):6025. 10.3390/ijerph20116025 37297629 PMC10252486

[53] Muras A Mallo N Otero-Casal P Pose-Rodríguez JM Otero A. Quorum sensing systems as a new target to prevent biofilm-related oral diseases. Oral Dis. (2022) 28(2):307–13. 10.1111/odi.13689 33080080

